# Metabolic management of a successful pregnancy and postpartum complications in fructose‐1,6‐bisphosphatase deficiency

**DOI:** 10.1002/jmd2.12453

**Published:** 2024-10-21

**Authors:** Callie Ferguson, Anita Madison, Ada Hamosh, Celide Koerner

**Affiliations:** ^1^ McKusick‐Nathans Department of Genetic Medicine Johns Hopkins University Baltimore Maryland USA; ^2^ Division of Reproductive Endocrinology and Infertility, Department of Gynecology and Obstetrics Johns Hopkins University School of Medicine Baltimore Maryland USA

**Keywords:** breast feeding, FBPase deficiency, fructose, hypoglycemia, inborn error of metabolism, pregnancy

## Abstract

Fructose‐1,6‐bisphosphatase (FBPase) deficiency is a rare, inborn error of metabolism, that causes hypoglycemia and lactic acidosis in response to inadequate glucose intake and/or high intakes of fructose, sucrose, or sorbitol. Pregnancy in women with FBPase deficiency puts them at high risk for metabolic decompensation due to increased glucose demands from the growing fetus. Here we report a 31‐year‐old primipara who was treated starting at 14 weeks gestation with a diet high in complex carbohydrates and low in fructose, sucrose, and sorbitol and close monitoring of glucose levels throughout her pregnancy. She delivered a healthy 2860 g baby at 37 weeks via vaginal delivery with no complications or hypoglycemia. At 5 months postpartum and 5 months of life, the patient and baby are doing well, although the patient experienced an episode of hypoglycemia and lactic acidosis at 4 months postpartum due to the increased metabolic demands of breastfeeding. This report adds to the limited case reports that discuss outcomes and proposed interventions during pregnancy in individuals with FBPase deficiency.

**Synopsis**

A patient with fructose‐1,6‐bisphosphatase deficiency delivered a healthy baby with no complications but required hospitalization in the first trimester due to vomiting and postpartum due to increased metabolic demands of breastfeeding.

## 1 INTRODUCTION

Fructose‐1,6‐bisphosphatase (FBPase) deficiency is a rare, autosomal recessive disorder, with an incidence of 1:350 000 to <1:900 000.^1^ Deficiency in the FBPase enzyme disrupts gluconeogenesis making patients with this defect rely solely on glucose intake and the breakdown of hepatic glycogen to maintain normoglycemia. Gluconeogenesis is the metabolic pathway that produces glucose from substrates including lactate, glycerol, fructose, and gluconeogenic amino acids. FBPase irreversibly converts fructose 1‐6 bisphosphate to fructose 6‐phosphate and inorganic phosphate, and the lack of FBPase results in hypoglycemia, once glycogen stores have been depleted. FBPase deficiency typically presents in early childhood with findings of hypoglycemia, lactic acidosis, and ketonuria manifesting as apnea, seizures, severe hyperventilation, hypotonia, and moderate hepatomegaly. Symptoms are more common in infancy and early childhood due to a lack of significant glycogen stores.^2^ Other factors known to trigger an episode are fever, fasting, decreased oral intake, vomiting, infections, and ingestion of large amounts of fructose. Dietary management of FBPase deficiency is not well defined and differs among inherited metabolic disease centers. Most centers do recommend avoiding prolonged fasting and excessive intake of fructose and sucrose regardless of age; however, others restrict it only while ill or only in childhood. In adults, it has been reported that patients usually remain well between episodes of acute decompensation. In addition to fructose and sucrose restriction, some centers treat with cornstarch, Glycosade, or complex carbohydrates at bedtime.^3^

At the time of this report, two case studies have been published on outcomes for pregnant individuals with FBPase deficiency.^4^, ^5^ As pregnancy can cause decreased oral intake, vomiting, and increased energy needs, close follow‐up is imperative. This case study adds to the existing literature of a successful outcome of the metabolic management of a pregnant woman with FBPase deficiency. In addition to hypoglycemia seen during pregnancy, this patient also experienced hypoglycemia at 4 months postpartum related to increased demand of breastfeeding that has not been previously reported.

### 1.1 Case

A 31‐year‐old, primipara woman with a known history of FBPase deficiency, presented to the emergency department (ED) at 14 weeks gestation with persistent vomiting. Glucose in the ED was found to be 78 mg/dL (4.33 mmol/L) with a lactate of 3.8 mmol/L and moderate ketones on urinalysis. Prior to this episode, the patient reports being able to manage her nausea with doxylamine/pyridoxine (Diclegis™) and blood sugar with increased carbohydrate intake at home. On the day of admission, she was vomiting approximately once per hour and unable to keep any fluid or food down. In the ED, she was started on 10% dextrose solution at 250 mL per hour (GIR 5.8 mg/kg/min) which was later switched to 5% dextrose with normal saline at 200 mL per hour (GIR 2.3 mg/kg/min).

The patient was initially diagnosed with FBPase deficiency in early infancy after presenting with hypoglycemia and liver injury with hepatosplenomegaly. She was hospitalized about twice per year until age 5, at which time her disease severity lessened. She had three additional hospitalizations from age 5 through high school and two while in college. At age 27 she had one hospitalization while working for many hours straight followed by contracting gastroenteritis. She was followed by a separate clinic through childhood but had not been seen recently. As we were unable to obtain confirmatory testing from her prior clinic, sequencing and deletion/duplication testing were performed on the *FBP1* gene showing a homozygous pathogenic deletion of exon 1. The patient is of South Asian Indian descent and was born to third cousin parents.

Prior to becoming pregnant, the patient did not follow any disease‐specific diet when well. While ill she treated herself with frequent meals consisting of carbohydrates, limited her fructose intake, and had liquid glucose supplements to treat hypoglycemia. She did have a glucose monitor at home and monitored glucose when ill or with poor intake. The patient and her partner underwent preconception genetic counseling including 381 gene carrier screening. Her partner also had sequencing of the *FBP1* gene and was found to not be a carrier for this condition.

As this was the patient's first pregnancy, conservative management was recommended. Recommendations were to use a continuous glucose monitor, start a fructose‐ and sucrose‐limited diet, eat frequent meals containing complex carbohydrates, and take Glycosade overnight or during other prolonged periods of fasting. During her pregnancy and while breastfeeding, the patient took 325 mg ferrous sulfate (65 mg elemental iron) and a prenatal multivitamin. She was also prescribed Zofran and Compazine as needed for nausea.

The patient's blood sugar remained stable throughout the remainder of the pregnancy after the initial hospitalization, with report of fasting blood glucose of 80s‐low 100 s mg/dL (4.5–6 mmol/L), and post‐prandial 140–150 mg/dL (7.8–8.3 mmol/L). The patient did not use Glycosade overnight. She used Glycosade a few times during the day while working and was unable to take breaks for carbohydrate intake. She had no further vomiting during pregnancy or any evidence of metabolic decompensation. The patient's prepregnancy weight was 70.5 kg with a BMI of 25 kg/m^2^. At the time of delivery, the patient was 81.9 kg for a total weight gain of 11.4 kg during pregnancy. Recommended weight gain by the patient's BMI is 11.4–15.9 kg. Fetal growth during pregnancy was appropriate measuring between 60%–81% estimated fetal weight at growth ultrasounds at 21, 29, and 33 weeks gestation.

The patient was induced at 37 weeks due to gestational hypertension and gave birth to a healthy boy 1 day later weighing 2860 g. During labor, she was maintained on 10% dextrose with half normal saline and 20 mEq/L KCl at two times maintenance. To induce labor she was given 25 mcg misoprostol, 1–30 milli‐units/min Pitocin, and an epidural. After delivery, fluids were slowly weaned with blood glucose checks every 4 h with goal of no lower than 80 mg/dL (4.44 mmol/L). The baby's blood glucose at birth was 73 mg/dL (4.06 mmol/L). The baby was not screened for FBPase deficiency at birth as the patient's partner was known to not be a carrier for this condition.

On Day 5 of life, the patient's newborn presented to an outside hospital due to poor intake, lethargy, and hypothermia with concern for sepsis. He was then transferred to our hospital for neonatal intensive care unit admission. Hypotension improved with fluid resuscitation and lactate was found to be normal. With adequate fluid and nutritional intake, the neonate was discharged home 2 days later.

At 4 months postpartum, the patient was admitted to an outside hospital after experiencing dizziness and was found to have a point‐of‐care glucose below 50 mg/dL (2.78 mmol/L). Patient reported having breakfast and lunch during the day and no other symptoms aside from having diarrhea the week prior that had resolved. After becoming hypoglycemic, she did have one episode of vomiting. In the ED, she had a glucose of 46 mg/dL (2.55 mmol/L), bicarbonate of 15 mmol/L, anion gap of 26 mmol/L, and lactate of 8.9 mmol/L. She was started on a 10% dextrose infusion at 200 mL per hour with improvement in acidosis. Aside from the two hospitalizations (at 14 weeks gestation and 4 months postpartum) as well as during delivery, the patient did not require IV dextrose during pregnancy. As the patient's glucose had been stable, she stopped using a continuous glucose monitor a few months after delivery. After the hospitalization, we recommended restarting the continuous glucose monitor and using Glycosade as needed to assist with blood glucose control. glycosade was only used a few times when her demanding job prevented her from eating frequent meals. The trigger for hypoglycemia was thought to be related to increased metabolic demands due to breastfeeding without a proportional increase in the patient's caloric intake.

## 2 DISCUSSION

Treatment of FBPase deficiency results in frequent hospitalizations in childhood; however, as patients get older, hospitalizations decrease.^6^ Dietary treatment of FBPase deficiency varies greatly, with some metabolic centers treating with a fructose and sucrose‐limited diet only when sick. Other centers treat with a limited fructose and sucrose diet at all times and restrictions do not relax with age. Pinto et al.^3^ found that 20 centers (56%) reported the use of cornstarch for FBPase deficiency. Pregnancy adds a unique stressor to individuals with FBPase deficiency because of the increased risk of a crisis without the close monitoring and recommendations from a metabolic team. Hypoglycemic events during pregnancy may be caused by poor maternal reserves of hepatic glycogen, inadequate glucose intake, and increased glucose needs due to the stress of pregnancy and growing demands of the fetus.^4^ Hypoglycemia and lactic acidosis pose risks of poor fetal growth and possible fetal death.^5^ There are few case reports available about outcomes of pregnancy, and those that exist have variable outcomes. In 2007, Krishnamurthy et al.^4^ described a woman who delivered three healthy babies however she developed sensorineural hearing loss and early‐onset cognitive impairment after the birth of her last child. She initially presented during the second trimester of her first pregnancy with profound hypoglycemia and lactic acidosis. After the diagnosis was made, she was compliant with dietary management including nocturnal cornstarch. All three children were born and remained healthy through the newborn period. A case report in 2010 described four individuals from two Swedish families with FBPase deficiency, with one individual having two healthy children after uneventful pregnancies.^7^ Another case by Sugita et al.^5^ in 2014 discussed a patient who never experienced metabolic acidosis during pregnancy. She was hospitalized three times throughout pregnancy due to inability to tolerate food by mouth but was promptly started on 10% dextrose solution 1000–2000 mL per day and did not become hypoglycemic. At 2 years postpartum, she had no neurological abnormalities, and her child was developmentally normal. Similarly, our patient did require two hospitalizations, but neither she nor her son have experienced further complications at 5 months postpartum. The patient's episode of hypoglycemia and acidosis at 4 months postpartum while breastfeeding illustrates the importance of continued close monitoring postpartum, especially while breastfeeding. As the patient's blood sugars had been stable after the first trimester and were stable for the first few months postpartum, she decided to remove her continuous glucose monitor. From this experience, future recommendations would be to continue glucose monitoring throughout the duration of breastfeeding.

There have been 61 pathogenic variants identified to cause FBPase deficiency. A large deletion affecting exon 1 has been previously described in two Turkish families who are unrelated.^8^ No specific phenotypic presentation was reported. Patients with two copy number variants were found to be more likely to present with hepatic steatosis than other variants.^9^ Sakuma et al.^10^ have classified missense mutations into three functional categories and found a key role of protein misfolding in the pathogenesis of FBPase deficiency. To our knowledge no such classification exists for exon deletions.

While our patient was well, we did not limit her intake of vegetables but did advise her to limit fruit intake as much as possible. She was typically consuming one serving of fruit per day, estimated to contain 10–20 g of fructose in one sitting, which is below the recommendation of <1 g/kg at one time.^11^ Sucrose was not formally restricted however the patient followed a relatively low‐sugar diet at baseline. Her diet was also rich in complex carbohydrates at each meal. While ill, the patient reports sugary foods and high fructose foods are not appetizing therefore they were unintentionally restricted. It appears small amounts of fructose and sucrose are well tolerated during pregnancy while well. The most important recommendation is to avoid prolonged fasting and to receive intravenous dextrose if unable to ingest enough calories orally. Pregnancy poses an increased risk of a catabolic state, with increased surveillance necessary to prevent unfavorable outcomes for the patient and fetus. As with any inborn error of metabolism, a multidisciplinary team is essential to the care of patients with FBPase deficiency. Formal guidelines for the management of this condition are needed however difficult to establish for this very rare condition. Our patient was followed in clinic monthly by the metabolic dietitian and the patient was discussed routinely with the biochemical geneticist and maternal fetal medicine specialist. Although Glycosade is not approved for use in conditions other than Glycogen Storage Disease, it was found to be useful for our patient when regular access to food was limited by a demanding job with long work hours. Liver ultrasounds were not completed during the patient's pregnancy; however, as increased risk of hepatic steatosis has been found in patients with FBPase deficiency a lifelong liver monitoring protocol should be followed.^1^

## 3 CONCLUSION

This case report adds to the existing literature for the management of a safe pregnancy and delivery for a person with FBPase deficiency. We demonstrate that hypoglycemia and lactic acidosis are preventable with the use of a diet high in complex carbohydrates with limited amounts of fructose, continuous glucose monitoring, and Glycosade when long periods of fasting are unavoidable. Careful metabolic monitoring should continue while breastfeeding as energy needs remain high. An emergency protocol is essential for quick treatment of any metabolic decompensations. A multidisciplinary team including a biochemical geneticist, metabolic dietitian, genetic counselor, and an obstetrician trained in high‐risk pregnancies is essential to the care of these patients. We recommend monthly follow up with the metabolic dietitian throughout pregnancy to carefully monitor adequacy of intake and blood glucose control. Comprehensive guidelines are needed for better future management of patients with FBPase deficiency during pregnancy.

## AUTHOR CONTRIBUTIONS

CF and AH conceived the study. CF and CK were involved in data collection. CF drafted the article. All authors made contributions to writing and reviewing of the final article.

## CONFLICT OF INTEREST STATEMENT

Callie Ferguson, Anita Madison, Ada Hamosh, and Celide Koerner declare that they have no conflicts of interest.

## ETHICS STATEMENT

All procedures followed were in accordance with the ethical standards of the responsible committee on human experimentation (institutional and national) and with the Helsinki Declaration of 1975, as revised in 2000 (5).

## INFORMED CONSENT STATEMENT

Informed consent was obtained from all patients for being included in the study.
